# Global research hot spot and trends in tinnitus treatment between 2000 and 2021: A bibliometric and visualized study

**DOI:** 10.3389/fneur.2022.1085684

**Published:** 2023-01-04

**Authors:** Tao Ye, Kefan Chen, Dongyang Li, Kailong Yin, Yuan Li, Jin Long, Lian Hui

**Affiliations:** ^1^Department of Otorhinolaryngology-Head and Neck Surgery, The First Hospital of China Medical University, Shenyang, Liaoning, China; ^2^Department of Thyroid Surgery, The First Hospital of China Medical University, Shenyang, Liaoning, China; ^3^Department of Pancreatic-Biliary Surgery, The First Hospital of China Medical University, Shenyang, Liaoning, China

**Keywords:** tinnitus, management, bibliometric analysis, visualized maps, research frontiers

## Abstract

**Background:**

In the 21^st^ century, the prevalence of tinnitus is increasing, impacting approximately one in five people. It is a very complicated condition that significantly affects quality of life. Despite the availability of hundreds of tinnitus treatment options, none are very successful. In light of this, there has been a steady increase in studies on tinnitus treatments in the recent past. To comprehend them better, this study used bibliometric approaches to analyze and summarize 21^st^ century scientific research accomplishments in tinnitus treatment.

**Methods:**

The Web of Science Core Collection (WoSCC) was searched for papers that had been published and related to the treatment of tinnitus. VOSviewer, CiteSpace, R, and Tableau software programs were used to conduct bibliometric studies. To evaluate and visualize the results.

**Results:**

2,933 publications on tinnitus treatment were found in 74 countries. Between 2000 and 2021, publications increased steadily. Otolaryngology-Head & Neck Surgery had the highest impact factor, whereas Otology & Neurotology had the most magazines and the highest h, g, and m index. Langguth B was the most prolific author in terms of productivity during the past 21 years. Numerous eminent authors and organizations from multiple nations collaborated. With 626 papers, the United States of America (USA) contributed the most to this field, making them the leading contributor. Neuroplasticity, sound therapy, and cognitive behavioral therapy (CBT) have attracted the attention of researchers, leading to the development of innovative diagnostic and treatment strategies for tinnitus.

**Conclusion:**

This bibliometric study provides a comprehensive analysis of worldwide publications, cooperation, and research hotspots in tinnitus therapy, revealing the present status of research on this issue and guiding tinnitus treatment research in the coming years.

## Introduction

Tinnitus is the perception of sound in the absence of external auditory stimulation. Tinnitus is derived from the Latin word tinnire, which means “to ring” (1). The prevalence of tinnitus ranges from 10 to 15%, making it a prevalent disorder (2). Tinnitus can strain patients tremendously and drastically reduce their quality of life. Numerous tinnitus patients describe symptoms such as frustration, annoyance, irritability, anxiety, sadness, hearing issues, hyperacusis, insomnia, and concentration difficulties (3). It is anticipated that tinnitus will continue to rise in the 21^st^ century due to the fast-paced lifestyle, demographic shifts, and increased occupational and recreational noise exposure (4).

However, not all patients are required to or will be treated. Tinnitus is classified as acute (several days to 3 months), subacute (3 to 6 months), and chronic (more than 6 months) according to the time of onset. The majority of acute and subacute tinnitus may improve spontaneously (5). In a cohort study (6), approximately fifty percent of patients with severe tinnitus (moderate intensity, sleep difficulties, or both) improved after 5 years, with 43 percent of the improved patients reporting total remission and fifty-seven percent having relatively mild symptoms. In another study (7), eighty-two percent of individuals with tinnitus at baseline maintained persistent tinnitus 5 years later, showing a spontaneous improvement rate of approximately twenty percent. Similarly, subjects assigned to a “waiting list” control group in certain clinical trials showed a modest but significant reduction in tinnitus distress (8). The greatest spontaneous improvement was observed in patients with a shorter duration of tinnitus, a younger age, and a longer interval between assessment and follow-up.

Numerous attempts have been made to treat or even cure tinnitus (9), yet no treatment or intervention provides a completely satisfactory solution. Given the complexity and heterogeneity of tinnitus, it is likely that single-factor approaches will have only modest effects (10). Tinnitus treatment should be based on a comprehensive diagnosis of the etiology and concomitant aspects of tinnitus in a particular individual. Numerous effective therapeutic interventions are available today for reducing tinnitus severity (11). There are specific treatment options for certain subtypes of tinnitus. CBT alone or in conjunction with transcranial magnetic stimulation (TMS) can effectively reduce tinnitus. In addition, the American Clinical Practice Guidelines for Tinnitus (12) continue to recommend hearing aids, health counseling and education, cognitive behavioral therapy, and auricular therapy as the primary treatment strategies (13). The Guideline does not recommend drugs, diet, or neuromodulation techniques due to the lack of medical evidence supporting the use of drugs and neuromodulation. Recently, A Multidisciplinary European Guideline for Tinnitus only recommends CBT as the primary treatment strategy, and cochlear implantation is recommended for tinnitus patients with hearing loss (14). In general, the treatment of primary tinnitus involves a wide range of options, and there is no unified and effective treatment plan because its pathogenesis remains unknown.

As there was no comparable bibliometric analysis in the research field of tinnitus treatment, this paper provides the direction and reference for future research on tinnitus treatment by systematically and objectively evaluating the research foundation, frontiers, and focus on tinnitus treatment using similar tools.

## Methods

### Data sources

On May 22, 2022, we retrieved online from the Web of Science Core Collection (WoSCC) database. To find studies published between 2000 and 2021, we utilized the following keywords: TS = tinnitus AND TS = treatment. Titles, keywords, authors, institutions, countries, references, abstracts, and journals were all collected for each paper. Articles were included if they met the following criteria: (1) they covered the period from 2000 to 2021, (2) the article was indexed in WoSCC, and (3) on tinnitus treatment. It is important to note that the following materials were not considered: (1) meeting records, letters, procedures, amended articles, and duplicated articles; (2) non-English articles; (3) irrelevant literature, and (4) documents that are unpublished and lack adequate information for further investigation.

### Data analysis

Experts use bibliometric analysis to statistically examine all the resources contributing to a field of study (15). Qualitative and quantitative research trends may be evaluated by bibliometrics using the tools provided by literature databases. It may assist academics in assessing the value of various publications, organizations, and nations in a given area of study and in gauging the general trajectory of that subject's growth. As such, it may be used as a springboard for the creation of clinical recommendations in the area of tinnitus therapy (16, 17). This paper aims to 1) identify and study the characteristics of articles on tinnitus treatment, 2) summarize the current research results in this field, and 3) understand the research direction and hot spots in this field to provide a reference for future research directions (18). It can depict the findings of a literature review with a clear knowledge graph, which may ease data interpretation, make the result more comprehensive, and assist in discovering the internal relationships between the various pieces of information.

We utilized CiteSpace 6.1. R3 and VOSviewer 1.6.18 to analyze and display the authorship, affiliation, publication location, and keyword networks, respectively. In addition, author and literature citation analyses were carried out to produce useful visual maps. In addition, bursts of keywords were detected so that new keyword research could be carried out. In addition, we completed an analysis of the information contained within each cluster. A data perspective presented as a timeline may illustrate how the dominant patterns in a field have shifted over time. Rstudiov2022.07.2 was used to extract diverse data and generate statistical graphics for easy comprehension. At the same time, the software is used to compare the data exported from the WoSCC database with articles local and global citation scores, journal impact factor, JCR quartile, g, h, m indexes, categories, National publications, local and global citation scores, and authors' g, h, and m indexes were analyzed and summarized. Using Tableau v19.4.4, a globe map depicting the number of publications in each nation and area was developed. Each has its benefits and can play a complementary role. The CiteSpace data standardization method is based on set theory to the similarity measure of the knowledge unit. Within the time slice of the similarity algorithm, the time zone and timeline are outlined on the time dimension of the knowledge evolution process and the history of literature in a cluster span to comprehend the development process and trend of the area. VOSviewer employs a data standardization method based on probability theory and offers a variety of visual views in the fields of keywords, co-organizations, and coauthors, including network view, superposition view, and density view. It has the exceptional qualities of simple drawing and beautiful images (19).

## Results

**Figure 1:**
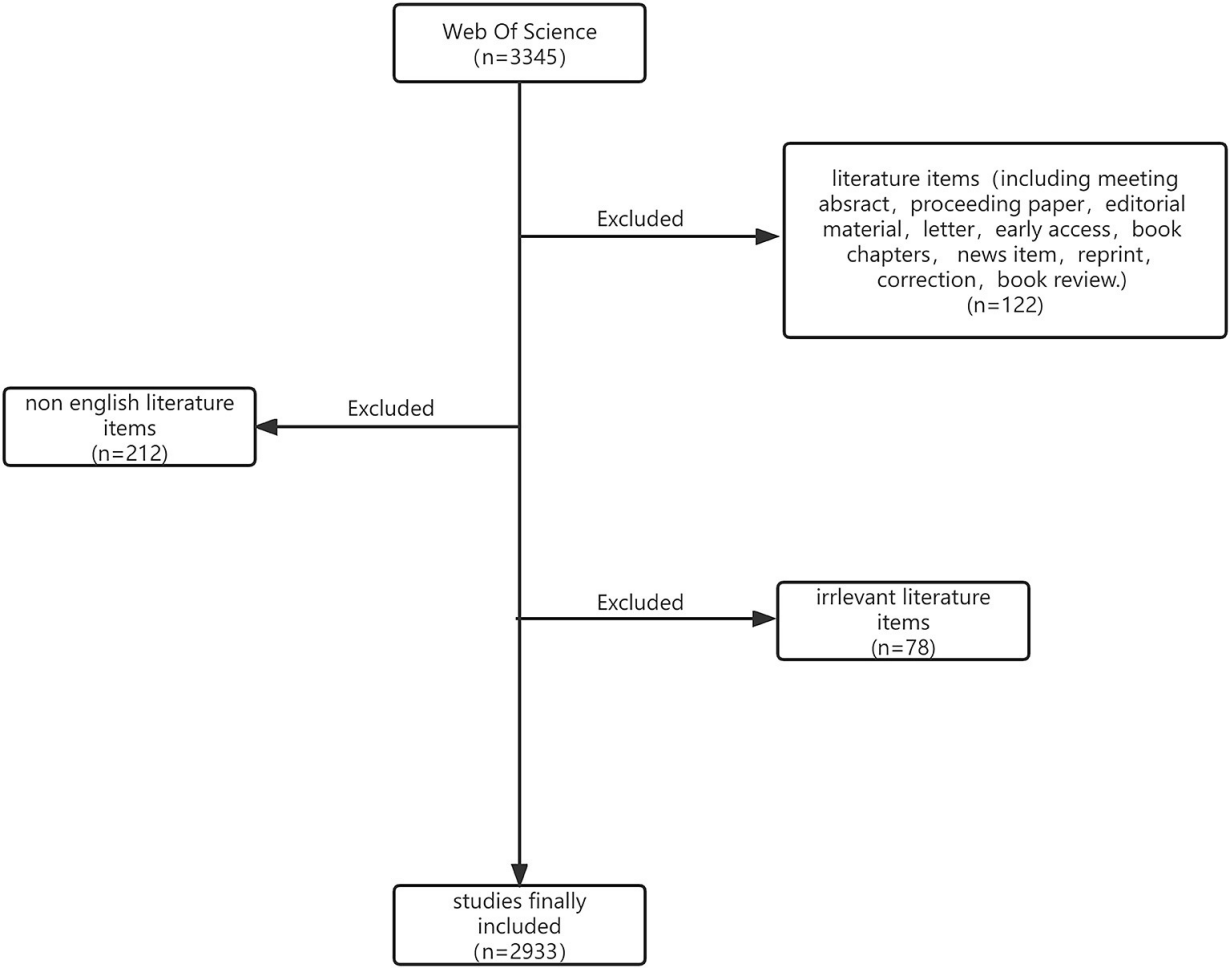
Flow diagram of the included papers.

**Figure 2:**
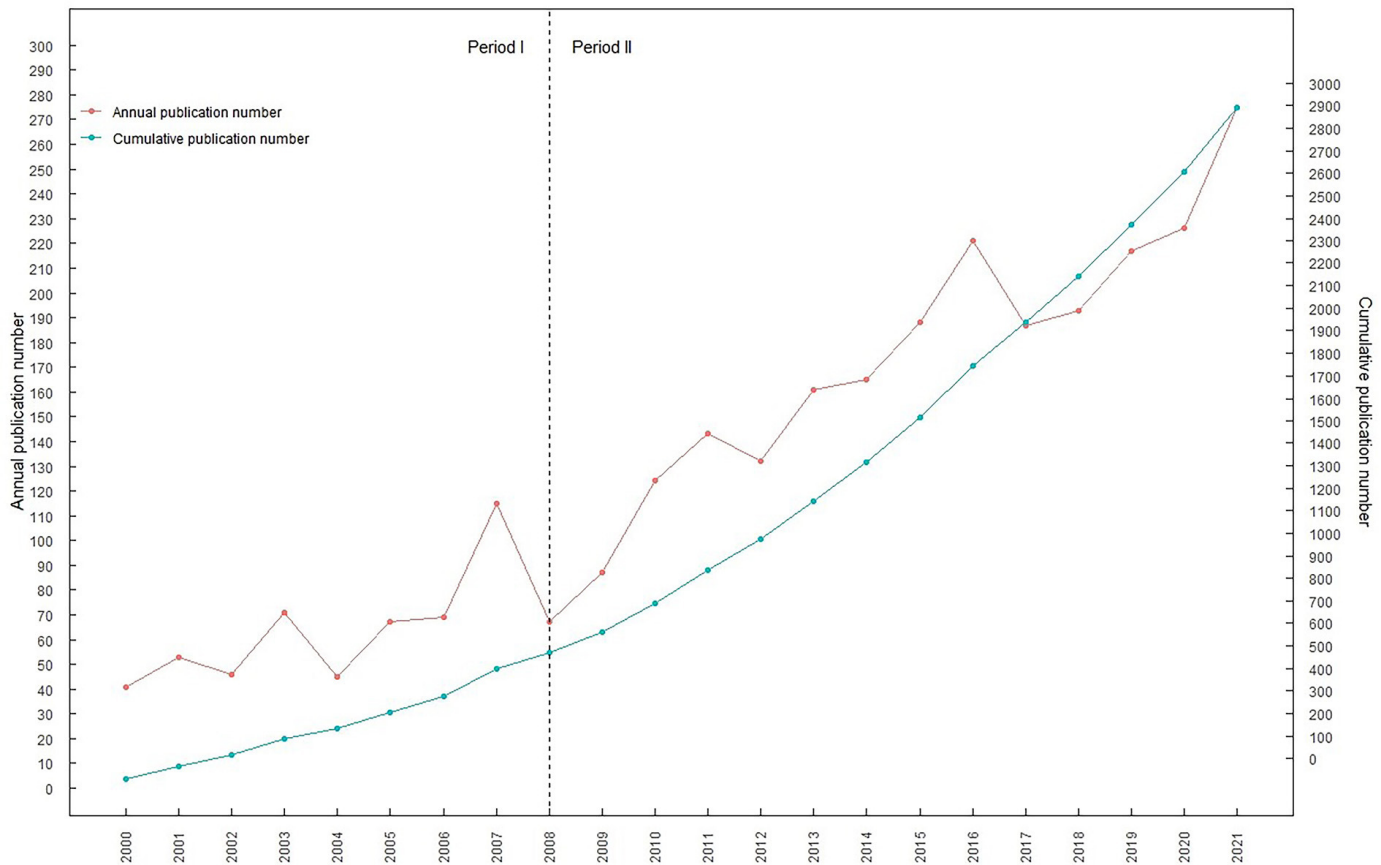
Publications of publications in tinnitus treatment research from 2000 to 2021. The red curve represents the annual publication number and the blue curve represents the cumulative publication number.

In total, 2933 documents fulfilled the requirements for retrieval (Figure 1). The annual output and overall output of publications are shown in Figure 2. The number of articles published per year can be divided into two phases in the 21st century: the first period of stable growth occurred from 2000 to 2008, and the second period of high increase will occur from 2008 to 2021. The total number of papers exhibited an increasing tendency from year to year, showing that the topic of tinnitus therapy research received growing focus. The number of articles published per year can be divided into two phases in the 21st century: the first period of stable growth occurred from 2000 to 2008, and the second period of high increase will occur from 2008 to 2021. As the numbers show, 2933 articles have mentioned a total of 61,074 times, with each piece referenced an average of 20.82 times.

### Leading journals and highly cited publications

Academic journals are the medium for the exchange and dissemination of information in a variety of fields. In total, 723 academic journals have published articles about tinnitus treatment research. The 20 core journals were determined based on Bradford's rule, particularly on the top 10. Table 1 lists the top 10 publications that have contributed the most to research on tinnitus therapy and their associated information. Otology & Neurotology, as the leading journal, published the most articles, next to the International Journal of Audiology, American Journal of Otolaryngology, Acta laryngologicalgica, Hearing Research, European Archives of Otorhinolaryngology, Laryngoscope, PLOS One, Otolaryngology-Head and Neck Surgery and Journal of Laryngology and Otology. Otolaryngology & Neurology journals make up the majority of this list. Most are in Q1 and Q2 of the Journal Citation Reports (JCR) quartile, and their 2021 impact factors are mentioned in Table 1. Most are higher than the previous year. Among them, otolaryngology-head and neck surgery belongs to JCR subregion Q1 and has the highest impact factor (5.591). Similarly, Table 1 reveals that the h index, g index, and m index of the journals Otolaryngology & Neurology, Hearing Research, and International Journal of Audiology are greater than those of other journals. All of the above are highly regarded academic publications that help scholars talk about tinnitus therapy and will, without a doubt, make a big difference in the growth of the field in the future.

**Table 1:** The leading journals that published publications in the tinnitus treatment field.

	**H_index**	**G_index**	**M_index**	TC^*^	NP^*^	**JCR quartile**	**IF^2021^**	**Categories**
Otology and neurotology	12	17	2	386	40	Q2	2.619	•Multidisciplinary sciences
International journal of audiology	10	14	1.67	252	29	Q2	2.437	•Medicine, research and experimental; otorhinolaryngology
American journal of otolaryngology	7	8	1.4	107	26	Q2	2.873	•Otorhinolaryngology
Acta oto-laryngologica	6	10	1	110	19	Q4	1.698	•Clinical neurology; otorhinolaryngology
Hearing research	10	13	1.67	220	18	Q1	3.672	•Audiology and speech-language pathology; neurosciences; otorhinolaryngology
European archives of oto-rhino-laryngology	7	13	1.17	171	14	Q2	3.236	•Otorhinolaryngology
Laryngoscope	7	13	1.17	179	14	Q2	2.97	•Otorhinolaryngology; surgery
Plos one	7	10	1.17	119	13	Q2	3.752	•Otorhinolaryngology
Otolaryngology-head and neck surgery	8	9	1.33	406	9	Q1	5.591	•Otorhinolaryngology
Journal of laryngology and otology	3	6	0.5	45	6	Q3	2.187	•Audiology and speech-language pathology; otorhinolaryngology

^*^NP, Number of published; ^*^TC, Total citations; ^*^IF, Impact factor.

Table 2 lists the characteristics of the highly cited articles, of which six correspond to the JCR partition Q1, two to the Q2 partition, and two to the Q3 division. “Evidence-based recommendations on the therapeutic use of repeated transcranial magnetic stimulation (rTMS)” was the most cited article from the 2014 edition of Clinical Neurophysiology. The paper with the greatest impact factor, “Tinnitus,” was published in the Lancet in 2013, greatly aiding research into tinnitus therapy. Six of the ten publications explored the effects of TMS (2, 12, 20), CBT(2, 12, 21) and sound therapy(2, 12, 22) on tinnitus treatment; discussed the varying impacts of these treatment measures on various types of tinnitus; and offered a solid reference for future study in the area of tinnitus treatment. Tinnitus is highly heterogeneous, and the two studies(2, 21) with the highest impact factors both advocated customized therapy based on tinnitus categorization. In a publication (12), the topic of tinnitus classification and tinnitus mechanism was studied in depth, hoping that more targeted treatments would be developed based on the pathologic and physiological mechanisms of specific tinnitus symptoms.

**Table 2:** The characteristics of the most referenced and impactful top 10 classic papers in the field of tinnitus treatment.

**Publications**	LCS^*^	GCS^*^	**JCR quartile**	**IF^2021^**	**Journal**	**Year**
•Evidence-based guidelines on the therapeutic use of repetitive transcranial magnetic stimulation (rTMS)	37	1,104	Q2	4.861	Clinical Neurophysiology	2014
•Evidence-based guidelines on the therapeutic use of transcranial direct current stimulation (tDCS)	10	722	Q2	4.861	Clinical Neurophysiology	2017
•Guided Internet-based vs. face-to-face cognitive behavior therapy for psychiatric and somatic disorders: a systematic review and meta-analysis	1	584	Q1	79.683	World Psychiatry	2014
•Tinnitus	171	528	Q1	202.731	Lancet	2013
•The tinnitus functional index: development of a new clinical measure for chronic, intrusive tinnitus	181	416	Q1	3.562	Ear and Hearing	2012
•Tuning out the noise: limbic-auditory interactions in tinnitus	132	412	Q1	18.688	Neuron	2010
•General review of tinnitus: prevalence, mechanisms, effects, and management	138	403	Q1	2.674	Journal of Speech, Language and Hearing Research	2005
•Phantom percepts: tinnitus and pain as persisting aversive memory networks	133	392	Q1	12.779	Proceedings of the National Academy of Sciences of the United States of America	2011
•Classification and epidemiology of tinnitus	121	360	Q3	1.866	Otolaryngologic Clinics of North America	2003
•Clinical practice guideline: tinnitus	131	341	Q1	5.591	Otolaryngology–Head and Neck Surgery	2014

^*^LCS, Local citation scores; ^*^GCS, Global citation scores.

### Leading countries and organizations

**Figure 3:**
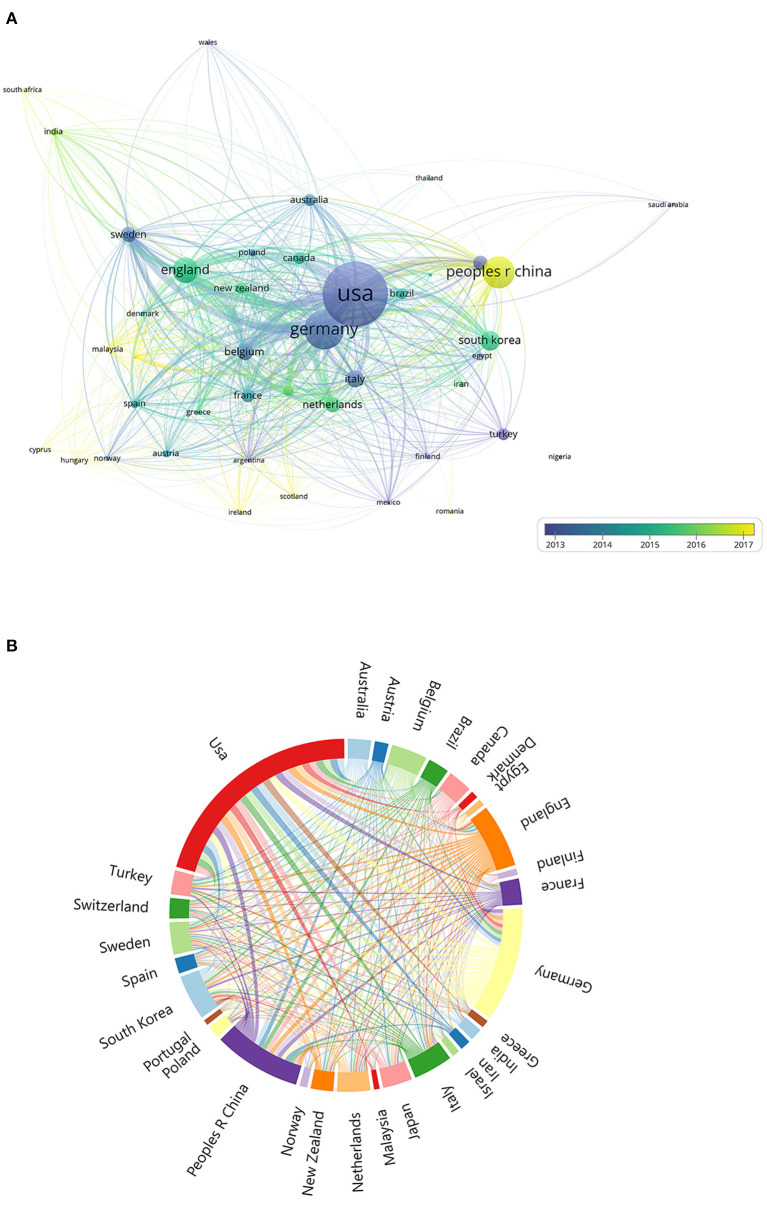
**(A)** The font size of each country/regions name represents the number of articles in the country/region. The thickness of the curved connecting line represents the collaborative intensity between countries/region; the country collaboration network of research on tinnitus treatment; different colors inside the circle represent different time intervals. **(B)** A chord chart of national collaboration. The thickness of the curved connecting line represents the collaborative intensity between countries/region.

**Figure 4:**
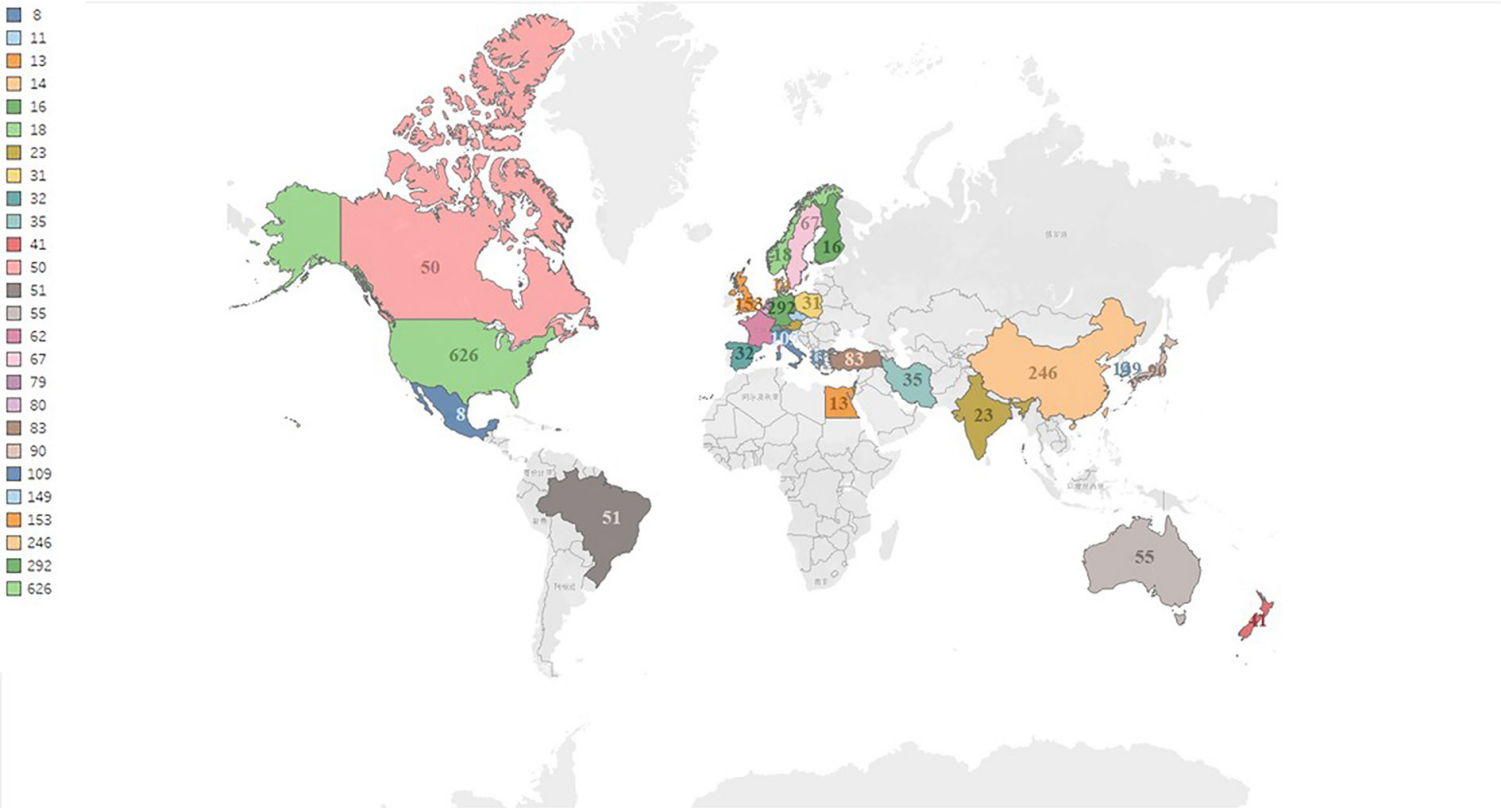
The global distribution countries on the research of tinnitus treatment.

In Figure 3, we can see the international research network on tinnitus treatments from 74 countries worldwide. The total number of publications from the top 30 nations was visualized on a global map (Figure 4). North America, East Asia, and Western Europe have contributed greatly to tinnitus treatment research. The top ten countries contributing to tinnitus treatment research are listed in Table 3. The United States (USA) contributed the most publications on tinnitus treatment, followed by Germany, China, the United Kingdom, Korea, Italy, Japan, Turkey, Belgium, and the Netherlands. The USA published the most articles alone (547 papers); however, Germany published more cooperation articles (83documentss) than the USA (79 pieces). Furthermore, Belgium's greatest MCP ratio shows extensive cooperation in this field. The top 10 nations accounted for 73.38% of global tinnitus treatment publications.

**Table 3:** Top ten countries in the tinnitus treatment field.

**Country**	**Publications**	**Publications proportion**	SCP^*^	MCP^*^	MCP_Ratio^*^	**LCS**	**GCS**
USA	626	24.09%	547	79	12.62%	4,932	27,596
Germany	292	11.24%	209	83	28.42%	3,891	16,790
China	246	9.47%	211	35	14.23%	540	3,230
United Kingdom	153	5.89%	103	50	32.68%	1,859	9,666
Korea	149	5.73%	133	16	10.74%	470	2,698
Italy	109	4.19%	92	17	15.60%	464	4,645
Japan	90	3.46%	84	6	6.67%	202	1,367
Turkey	83	3.19%	82	1	1.20%	183	937
Belgium	80	3.08%	52	28	35.00%	1,628	6,793
Netherlands	79	3.04%	55	24	30.38%	676	4,788

^*^SCP, Single country publications; ^*^MCP, Multiples country publications;^*^ MCP_Ratio, MCP/ Publications.

**Figure 5:**
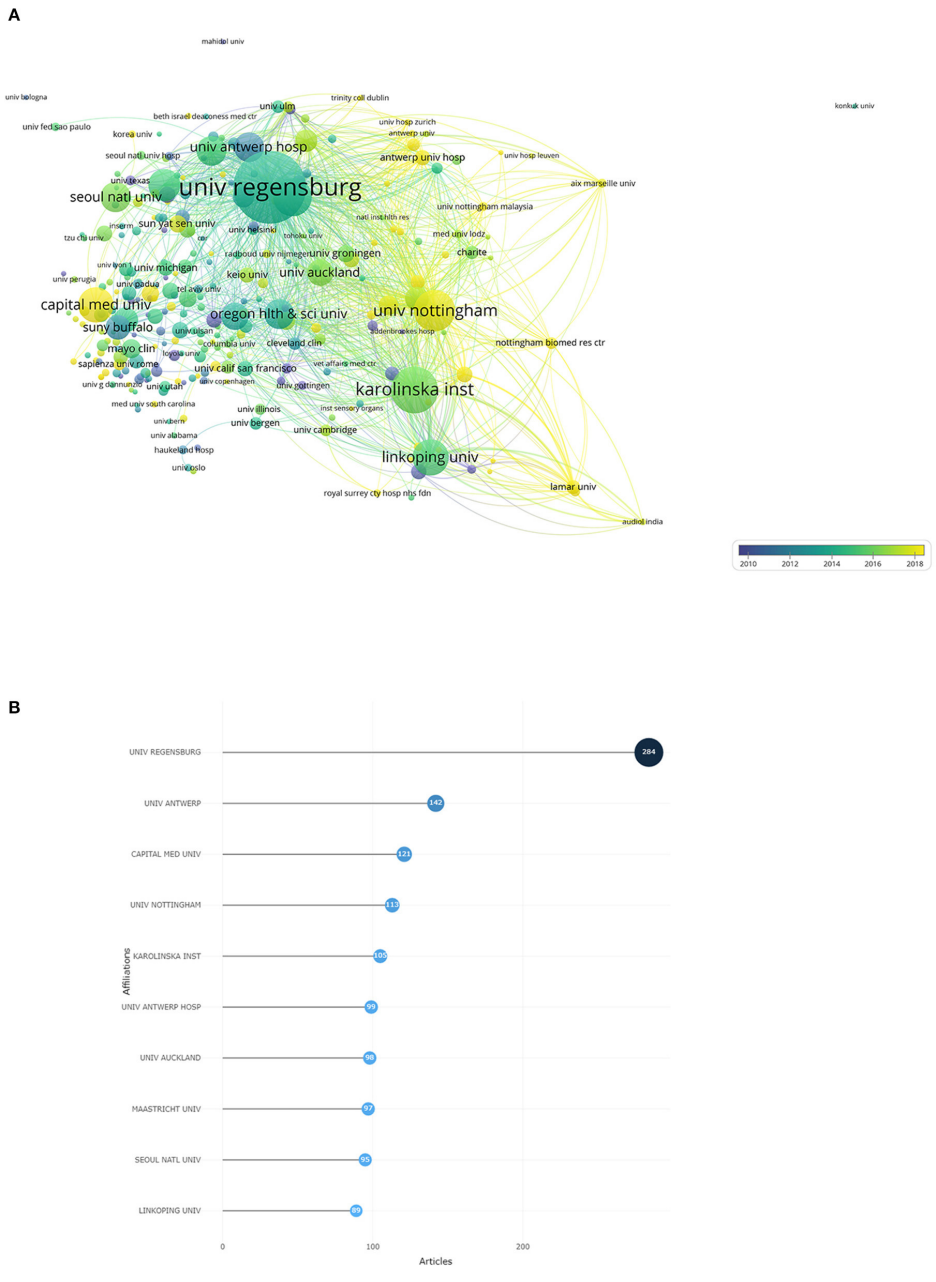
**(A)** The font size of each institution's name represents the number of articles in the institutions. The thickness of the curved connecting line represents the collaborative intensity between institutions; the institutions collaboration network of research on tinnitus treatment; different colors inside the circle represent different time intervals. **(B)** Top 10 research institutions on tinnitus treatment.

The institutional collaboration network of tinnitus treatment (Figures 5A,B) showed that the top ten most productive institutions were Univ Regensburg, Univ Antwerp, Capital Medical Univ, Univ Nottingham, Karolinska institution, Univ Antwerp Hosp, Univ Auckland, Maastricht Univ, Seoul Natl Univ, and Linkoping Univ. Univ Regensburg has published 284 articles on tinnitus treatment, more than any other institution.

### Authors and co-cited authors analysis

By analyzing the authors of the literature, we can identify the most influential researchers and scholars in the field of study. The top ten highly productive authors who have published more than thirty-four articles in this field are listed in Table 4.

**Table 4:** Top ten authors most frequently appearing in the publications.

**Author**	NP^*^	TC^*^	**H_index**	**G_index**	**M_index**
Langguth B	159	8,813	49	90	2.45
De Ridder D	86	6,277	37	78	1.947
Andersson G	65	3,758	32	61	1.455
Hajak G	40	2,828	30	40	1.5
Kleinjung T	52	3,079	30	52	1.667
Van De Heyning P	69	2,501	30	48	1.579
Landgrebe M	51	2,499	29	49	1.706
Vanneste S	65	2,813	28	52	2.154
Mazurek B	52	1,617	24	39	1.412
Henry JA	34	3,133	21	34	1.543

^*^NP, Number of published; ^*^TC, Total citation.

**Figure 6:**
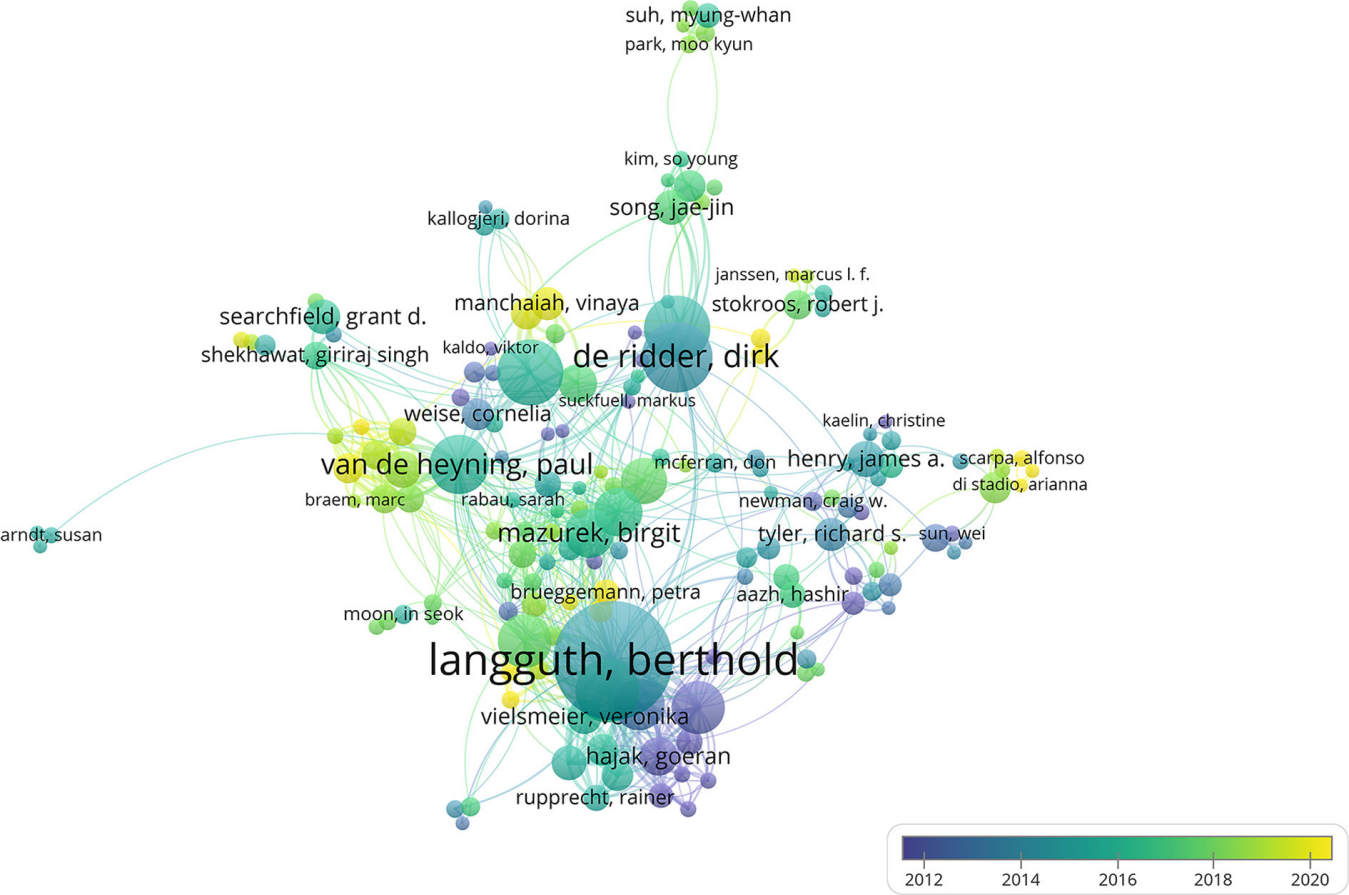
The font size of each author's name represents the number of articles. The thickness of the curved connecting line represents the collaborative intensity between authors; the authors collaboration network of research on tinnitus treatment; different colors inside the circle represent different time intervals.

The author's collaboration network on tinnitus treatment was observed (Figure 6). In terms of publications, Langguth B, De Ridder D, Andersson G, Hajak G, Kleinjung T, Van De Heyning P, Landgrebe M, Vanneste S, Mazurek B, and Henry Ja were the ten most prolific writers during the previous 21 years. In addition, the data on author TC were studied, which can be seen in Table 4. In terms of the number of TCs, Langguth B had the most citations; moreover, his h, g, and m indexes were greater than those of other authors, reflecting the highest academic authority level.

### Co-occurring keyword analysis

**Figure 7:**
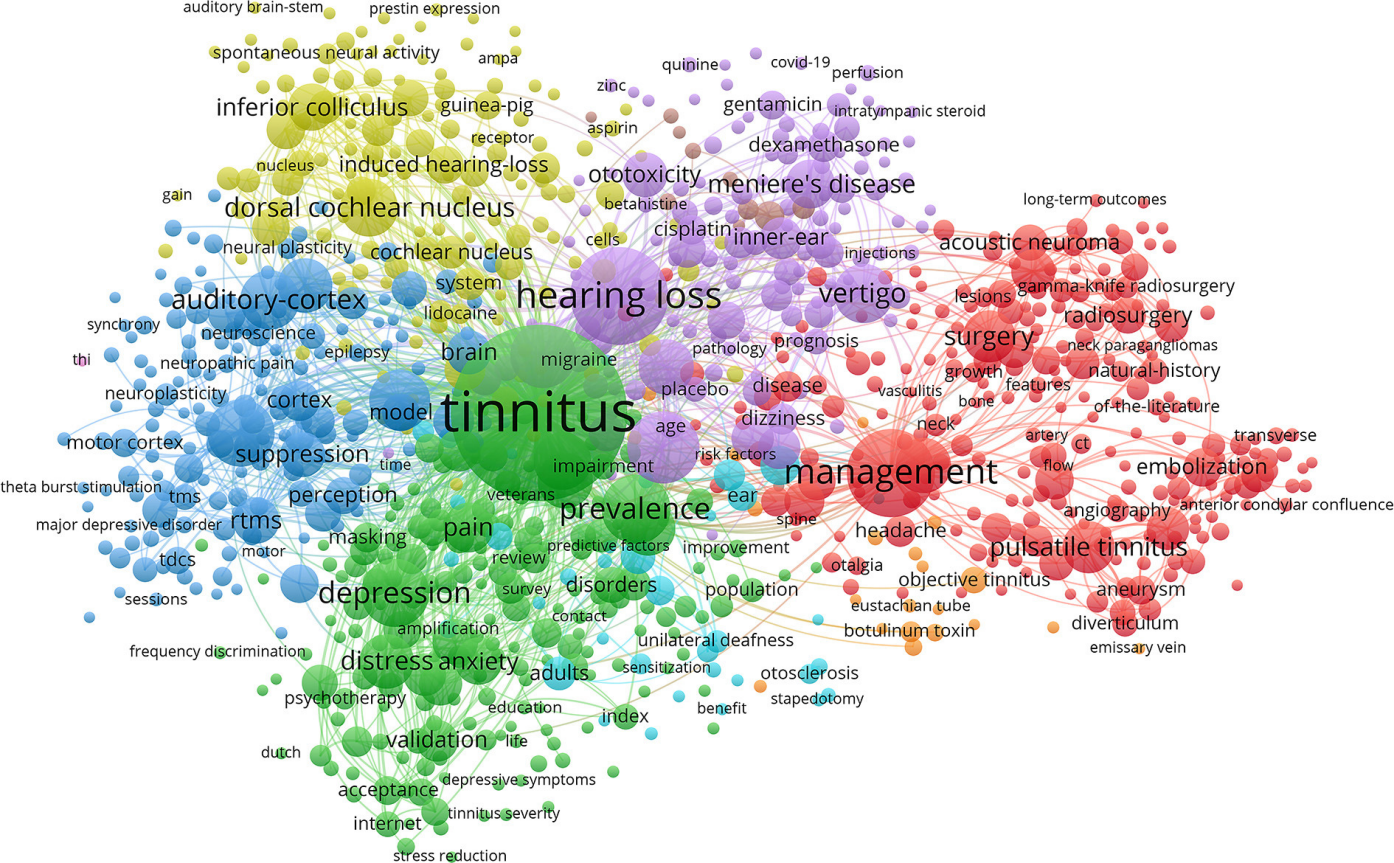
The font size of each keyword's name represents the number of articles in the institutions. The thickness of the curved connecting line represents the collaborative intensity between keywords; the keywords collaboration network of research on tinnitus treatment, and the same color represents the same cluster; the brighter the color, the more research about these keywords.

**Figure 8:**
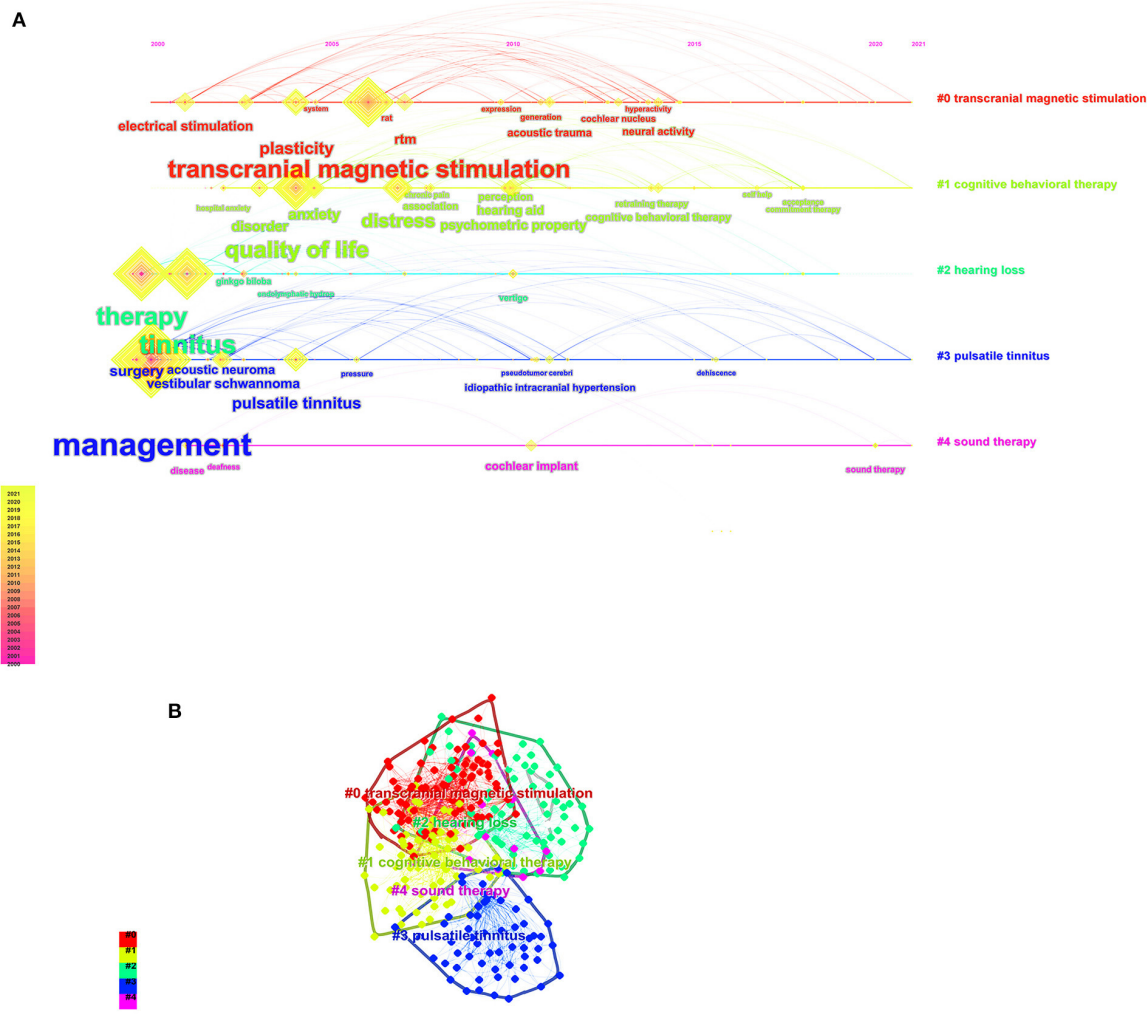
**(A)** The timeline view of the knowledge map in tinnitus treatment field. This view clearly presents the differences in the appearance time point and time span of five clusters. **(B)** Keyword cluster map for tinnitus treatment.

A total of 8,450 keywords were identified for the collaboration network of tinnitus treatment, as shown in Figure 7. Topics related to tinnitus (1,223), hearing loss (409), management (307), therapy (207), prevalence (189), depression (185), transcranial magnetic stimulation (181), mechanisms (147), auditory cortex (139), and vertigo (139) were the most discussed keywords. All of these terms were organized into six distinct clusters: cluster 1 was associated with “transcranial magnetic stimulation (TMS),” cluster 2 correlated with “hearing loss,” cluster 3 was linked to “pulsatile tinnitus,” cluster 4 was related to “cognitive behavioral therapy (CBT),” and cluster 5 was connected with “sound therapy.” The view of the timeline and cluster map from 2000 to 2021 is depicted in Figure 8, along with the evolution of the keywords over time for each cluster. The initial research focus was on “medical therapy, masking therapy, and acupuncture,” whereas the current focus was on “TMS, CBT, and sound therapy.”

### Burst terms analysis and frontier research

**Figure 9:**
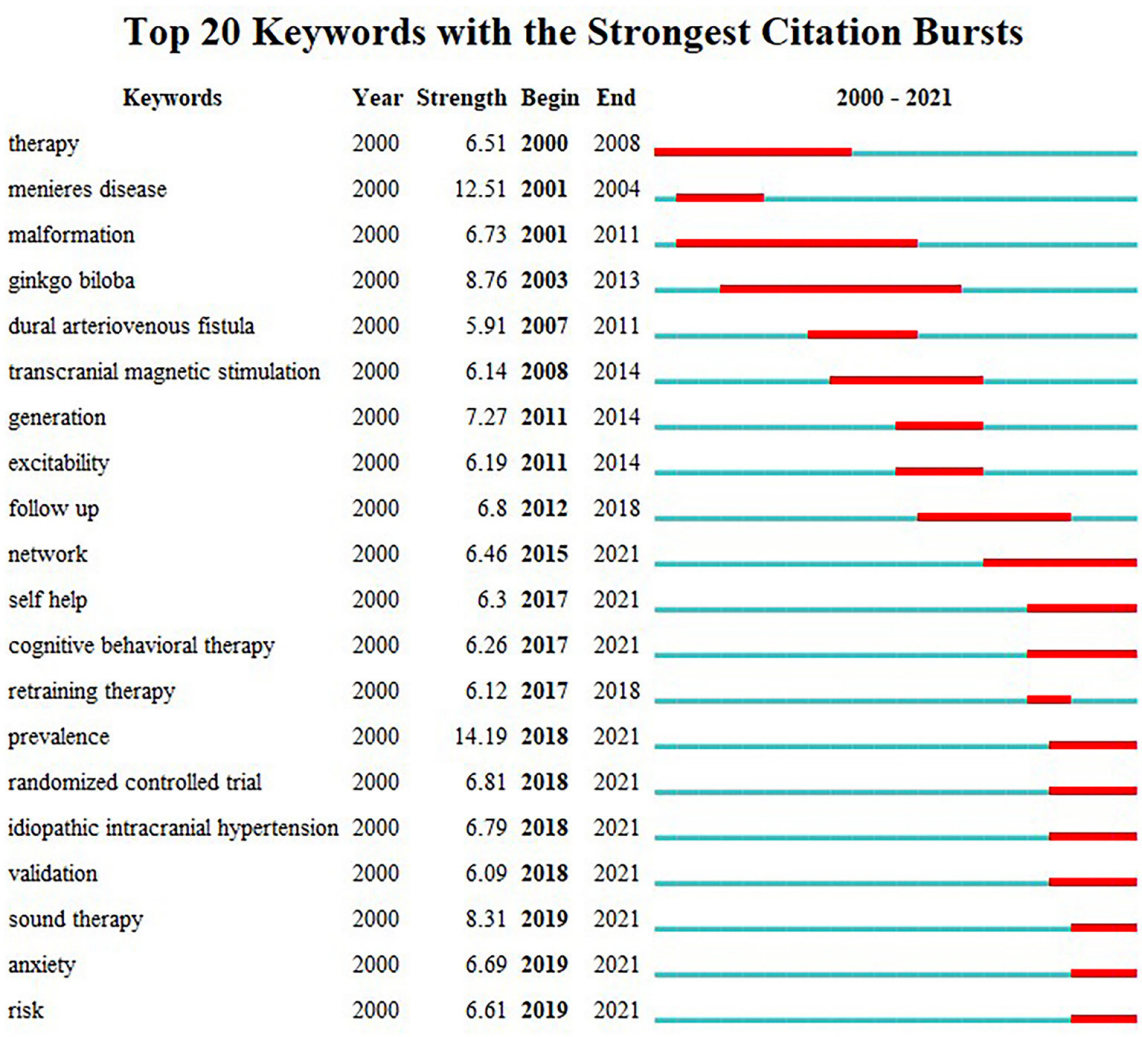
The top 20 burst words for tinnitus treatment.

In addition, the term burst is employed as a sensitive marker of the present research tendencies. We utilized CiteSpace to produce a term burst map (Figure 9) that illustrates the strength of the beginning and end. We identified the top 20 keywords with the strongest citation bursts r in the field of tinnitus treatment research. Among them, the five strongest burst terms are “prevalence,” “Meniere's disease,” “ginko biloba,” “sound therapy,” and “generation.” The following were the initial 21st-century research directions: “therapy” started in 2000, “Meniere's disease and malformation” began in 2001, “ginko biloba” started in 2003, “dural arteriovenous fistula” began in 2007, “transcranial magnetic stimulation” began in 2008, “generation and excitability” began in 2011, “follow up” began in 2012, “network” started in 2015, “self-help, cognitive behavioral therapy (CBT) and retraining therapy” began in 2017, “prevalence, randomized controlled trial (RCT), idiopathic intracranial hypertension and validation” began in 2018, and “sound therapy, anxiety and risk” started in 2019. The earliest burst term among them began in 2000, although most burst terms started after 2015.

## Discussion

The bibliometric and visualized research gives a complete overview of the growth of the academic literature from 2000 to 2021 concerning treatments for tinnitus. Therefore, relevant writers and research teams could clearly define the present state of research on this topic and the potential for future research in this field. This is the first-time bibliometric approaches have been used to research articles on tinnitus treatment obtained from 2,933 different WoSCC databases since many bibliometrics were initially discovered.

While the total number of publications in the United States, at 626, is the highest of any country, the number of individual publications, at 547, is also the highest. However, the number of joint publications is somewhat higher in Germany. Its h, g, and m indexes are above average compared to countries. Most top tests were developed in Asian, American, and European nations. There may be a correlation between the research output of these nations and their degree of cultural development, the number of researchers interested in this topic, and the level of financial assistance supplied to researchers.

As far as academic journals are concerned, there has been an increasing flow of articles on tinnitus therapy in the 21^st^ century. The vast majority of articles in this area reflect solid pieces of academic inquiry. There is a potential for researchers in this area to get widespread recognition thanks to the high average number of citations per document (20.82). Langguth B had the highest h-index, g-index, and m-index among the key authors who had written studies on tinnitus treatment, and he had the most publications published. However, it was discovered that the worldwide research teams had distinct geographical characteristics, with the majority coming from North America, East Asia, and Western Europe. Departments were concentrated in a university teaching hospital's otolaryngology and neurography divisions. Consequently, it is recommended that international research teams improve their lines of communication and work together to prepare for future findings.

There is a direct link between keywords and research topics in Figure 7. Tinnitus, management, hearing loss, transcranial magnetic stimulation (TMS), and depression were among the high-frequency keywords in our research. This suggests that the majority of s most studies have focused on tinnitus treatment. There are many ways to treat tinnitus, but few are very effective (23–26) The vast majority of these keywords could be found in the center of the network. They were significant as core phrases because they represent the topics of interest in this field (Figure 8) for the keyword, clusters display the development of each cluster displays. Cluster analysis classified the cluster's keyword terms into 5 clusters. They are beginning with the original study emphasis on “musical treatment, masking therapy, and retraining therapy” and progressing up to the current research for “CBT, sound therapy and TMS” (27). This provides evidence that the focus of research on tinnitus in recent years has shifted to investigations into new techniques for treating tinnitus.

The first cluster was associated primarily with TMS. Tinnitus is commonly caused by acute or chronic cochlear damage or diseases such as auditory trauma, drug toxicity, and presbyacusis. Its neural correlates reflect central changes caused by neuroplasticity accompanied by hypersynchrony or hyperactivity in cortical and subcortical auditory and non-auditory areas (28, 29). TMS was initially used to stimulate the primary motor cortex in humans in 1985 (30). The advent of central generation and maintenance theories for subjectively disabling tinnitus inspired the use of TMS for tinnitus treatment (31, 32). Recent research has focused on using low-frequency repetitive TMS in the temporal lobe or temporoparietal cortical regions on one side of the head to “quiet” the so-called lateralized hyperactive auditory cortex. Multiple novel TMS paradigms have been developed as extensions of these classic protocols to alter cortical excitability(33). Continuous “theta burst stimulation” (cTBS) is one of the most popular methods. A recent study likewise found a favorable effect of high frequency(HF) 10 Hz repetitive TMS applied to the left auditory cortex; however, cTBS applied bilaterally on auditory cortices was significantly more effective (34). In a separate study, cTBS of the auditory cortex decreased tinnitus, particularly its emotional component (35). Although repetitive TMS has effectively treated tinnitus, many questions remain unanswered, particularly in studies including long-term intervention. In brief, TMS can reduce tinnitus; however, its benefits are usually temporary and inconsistent (36, 37).

The second cluster focused on hearing loss (HL), accompanied by ringing in the ears. In many cases, tinnitus develops long after HL (38). HL and tinnitus are not always linked. Audiometry does not detect some forms of auditory deafferentation; therefore, not all patients with tinnitus have aberrant audiograms (39). HL is an important risk factor for many—though not all—tinnitus presentations (2, 40, 41). A formal audiology assessment is recommended for all patients presenting with tinnitus. This is because many patients with tinnitus have some degree of HL (42). Tinnitus is associated with many factors, including otological infections such as otitis media, acoustic pathway neoplasms, presbycusis, sensorineural hearing loss, noise exposure, neurological disorders, psychological disorders, ototoxic medications and so on (43); among these, HL is the main risk factor (44, 45). Eighty percent of those who experience sudden sensorineural hearing loss also experience tinnitus, which can be a precursor or contemporaneous symptom (46). These results suggest a correlation between the generation of tinnitus and damaged hearing.

The third cluster was connected with CBT for tinnitus. CBT is a brief psychological treatment that uses behavioral changes and cognitive restructuring to uncover and change maladaptive ideas and actions (47, 48). CBT has been used to treat tinnitus for 30 30 years, which was initially created to treat depression and anxiety and has been demonstrated to be beneficial in treating tinnitus-related discomfort (12). One study with a 15-year follow-up revealed the durability of treatment after therapy (49). Behavioral treatments are a part of the treatment. They consist of things like learning to relax, being exposed to things you're afraid of, establishing good sleep habits, and having your environment enriched with sounds. Hesser et al. evaluated 15 trials and discovered a sustained benefit of CBT on tinnitus-specific outcome measures and a clear role for CBT in alleviating depression and improving quality of life (50). In a UK guideline for tinnitus management, only CBT had enough data for statistical pooling to show efficacy (51). With the proliferation of the Internet, Internet-delivered CBT has become popular and attractive due to the prospect of increased access to such treatments. Both Internet-delivered CBT and group CBT led to comparable improvements in tinnitus immediately after posttreatment and 1 year later (52). In addition, Internet-based treatment is less expensive and shorter. The Cochrane meta-analysis of five RCTs found a substantial difference from controls (SMD 0.64; 95% CI 0.29–1.00; I^2^ = 0%). Individual RCTs reduced depression scores (effect sizes from 29 to 0.37, SMD 0.37; 95% confidence interval 0.15–0.59; I^2^ = 0%) (53). The Oxford Center for Evidence-Based Medicine's evidence levels places this form of CBT at ***Ia***.

The fourth cluster was linked to pulsatile tinnitus (PT). PT is a syndrome with multiple etiologies that can lead to Ethiopia with various causes, some of which pose severe hazards to neurologic, ocular, or aural health (54), and it is often unilateral unless the underlying vascular pathology is bilateral. Pulsatile tinnitus falls under the category of “physical tinnitus” and “somatic sound” since there is typically an actual physical sound source (55). However, PT is experienced by >10% of people with tinnitus (56). Unlike non-pulsatile tinnitus, the underlying cause of PT can be diagnosed in more than 70% of patients with a comprehensive evaluation (57). The leading cause of PT was a highly vascularized temporal bone tumor (16%), followed by normal venous fluctuations and anomalies (14%) and vascular stenosis (9%). Dural arteriovenous fistula, inflammatory hyperemia, and intracranial hypertension tied for the fourth position (each with 8%) (58). In addition, PT produced by raised intracranial pressure severely affects 65% of all individuals with PT caused by elevated intracranial pressure (59, 60).

The fifth cluster was related to sound therapy. The first-time sound therapy was utilized to treat tinnitus in 1976 when sound generators based on the principle of dispersion were introduced, and total masking of tinnitus with white noise became a clinical treatment. Sound therapy is a retraining therapy for tinnitus (12) that minimizes the contrast between tinnitus and ambient noises, encourages tinnitus habituation, and suppresses the impression of tinnitus or the response to tinnitus by using external sounds to produce clinical results (61–63). A 2010 meta-analysis proved that sound therapy during exposure to disruptive noise with and without hearing aids (64) might effectively treat tinnitus in frequently simultaneously detectable hearing loss or primarily central or psychologically induced hearing loss (65). In another trial (*n* = 56), sound therapy at the tinnitus frequency (40 Hz) reduced loudness better than other therapies (66). There is a dearth of large-scale randomized controlled trials that provide definitive evidence for the efficacy of sound therapy in alleviating tinnitus-related distress. Still, the aging body of evidence suggests that this treatment method is effective for many people, making it an area of intense study (66–71).

Although great progress has been made in the treatment of tinnitus in recent years, its mechanism is still a mystery. From the initial drug treatment to electrical stimulation, magnetic stimulation, and psychological and physiological comprehensive therapy, the treatment of tinnitus has been constantly updated and developed. At present, although TMS has achieved some efficacy, whether it has adverse reactions to the human body, and its long-term safety and effectiveness are still unclear, and further studies are needed. CBT and sound therapy have been shown to be effective in recent studies, so these treatments may be promoted for clinical use. In the future, more forms of CBT and sound therapy, such as internet-based CBT and personalized sound therapy, may be widely used in clinical practice.

This research demonstrates the dynamic growth process and the structural connection of pertinent scientific information *via* the atlas of scientific knowledge by methodically compiling the relevant literature on this subject. It has been suggested that researchers working in tinnitus treatment should follow the center of scientific research and focus on current research hotspots and that research institutions should strengthen the interchange and cooperation among one another to promote the academic development of this field of study.

## Limitations

The WoSCC database served as the source for the papers used in this study's analysis. The fact that the major most search publications on tinnitus therapies are included in this dataset does not exclude the possibility that other datasets, such as PubMed and Scopus, may provide more comprehensive coverage. This is one of the limitations of this work. The majority of the listed research could be useful for making references in upcoming updates. Different analyses might be derived from the same dataset at the same time. In addition, the findings of this study report are entirely predicated on CiteSpace 6.1. R3 and VOSviewer 1.6.18, and the algorithm used by the computer to solve issues, is not as clever as the algorithm used by the human brain, making the conclusions more susceptible to bias. In addition, basic science researchers were underrepresented due to the inclusion criteria of the authors. Despite these caveats, the study employs bibliometrics to describe and assess this topic for the reference of future researchers and to offer guidance for the future growth of this discipline by revealing the future research trends hotspots in this field.

## Conclusion

In summary, this article covers global research trends in tinnitus treatment. Otolaryngology-Head and Neck Surgery was the most referenced journal overall, while Otology & Neurotology was the most productive journal. The United States had the most publications and citations. In contrast, Germany's most collaborative publications and the top 10 nations were mostly located in North America, East Asia, and Western Europe, which substantially aided in the advancement of research. Therefore, countries with a poor academic climate should boost their scholarly research activities and increase their expenditures on scientific research. This would help shrink the gap that now exists. The University of Regensburg has contributed to a substantial amount of research in this field. In addition, in the 21^st^ century, the number of articles produced in this sector has dramatically increased. Extensive international collaboration occurs between nations, organizations, and individual writers. Current research hotspots include CBT, TMS, quality of life, acoustic treatment and depression, and new treatments for tinnitus will lessen the impact the condition has on sufferers' lives and may even make existing treatments more effective. Acoustic therapy, low-frequency repetitive transcranial magnetic stimulation and cognitive behavioral therapy alone or combined for tinnitus have recently attracted attention. The multidisciplinary treatment of tinnitus in the 21^st^ century, including otolaryngology, neurology and psychology, is becoming a hot trend of the times. The research will continue to achieve results, and our study will provide a powerful reference for tinnitus research and clinical treatment.

## Data availability statement

The original contributions presented in the study are included in the article/supplementary material, further inquiries can be directed to the corresponding author.

## Author contributions

This work was conceived by LH, JL, and TY. Data was collected and downloaded by TY, KY, YL, DL, and KC. The visualization work was performed and the manuscript was written by TY. LH, JL, KY, YL, DL, and KC helped to revise manuscript and proposed constructive opinions. All authors contributed to the article and approved the submitted version.
